# Oral acyclovir prophylaxis against herpes simplex virus in non-Hodgkin lymphoma and acute lymphoblastic leukaemia patients receiving remission induction chemotherapy. A randomised double blind, placebo controlled trial.

**DOI:** 10.1038/bjc.1984.138

**Published:** 1984-07

**Authors:** H. Anderson, J. H. Scarffe, R. N. Sutton, E. Hickmott, D. Brigden, C. Burke

## Abstract

Forty-one patients receiving remission induction chemotherapy with vincristine, adriamycin and prednisolone (VAP) for high grade lymphoma or acute lymphoblastic leukaemia were entered into a double blind, placebo controlled trial of oral acyclovir prophylaxis against herpes simplex virus (HSV) infection. The dose of acyclovir was 200 mg four times daily for the duration of chemotherapy (six weeks). Of the 40 evaluable patients, 20 were randomised to each arm. Prophylactic oral acyclovir significantly reduced the incidence of clinical HSV infection from 60% on placebo to 5% acyclovir (P less than 0.001), and the incidence of viral isolates from 70% on placebo to 5% on acyclovir (P less than 0.001).


*Br. J. Cancer* (1984), **50**, 45–49

### Oral acyclovir prophylaxis against herpes simplex virus in non-Hodgkin lymphoma and acute lymphoblastic leukaemia patients receiving remission induction chemotherapy. A randomised double blind, placebo controlled trial

H. Anderson[1], J.H. Scarffe[1], R.N.P. Sutton[2], E. Hickmott[3], D. Brigden[3] & C. Burke[3]

[1]*Dept. of Medical Oncology, Christie Hospital & Holt Radium Institute,* [2]*Dept. of Virology, Withington Hospital, Manchester, and* [3]*Wellcome Research Laboratories, Beckenham, Kent, UK*

**Summary** Forty-one patients receiving remission induction chemotherapy with vincristine, adriamycin and prednisolone (VAP) for high grade lymphoma or acute lymphoblastic leukaemia were entered into a double blind, placebo controlled trial of oral acyclovir prophylaxis against herpes simplex virus (HSV) infection. The dose of acyclovir was 200 mg four times daily for the duration of chemotherapy (six weeks). Of the 40 evaluable patients, 20 were randomised to each arm. Prophylactic oral acyclovir significantly reduced the incidence of clinical HSV infection from 60% on placebo to 5% acyclovir ($P<0.001$), and the incidence of viral isolates from 70% on placebo to 5% on acyclovir ($P<0.001$).

Herpetic infections are common in immunosuppressed patients with malignant diseases (Aston *et al.*, 1972; Casazza *et al.*, 1966; Lam *et al.*, 1981; Meyers *et al.*, 1980; Muller *et al.*, 1972), and may be life threatening. The oral ulceration and stomatitis caused by HSV may be associated with significant morbidity. In the past year eight of 25 (32%) patients with non-Hodgkin's lymphoma, admitted to our oncology ward with pyrexia when neutropenic, had virologically confirmed HSV infections. Intravenous acyclovir has been shown in randomised, placebo controlled trials to be effective in the treatment of HSV infection in immunocompromised patients (Chou *et al.*, 1981; Mitchell *et al.*, 1981; Wade *et al.*, 1982), and in the prophylaxis of HSV infection (Hann *et al.*, 1983; Saral *et al.* 1981). A report of oral acyclovir prophylaxis in bone marrow transplant patients has shown a reduced incidence of HSV in those patients treated with acyclovir compared with placebo, without significant toxicity (Gluckman *et al.* 1983).

The aim of this trial was to evaluate the efficacy of prophylactic oral acyclovir against HSV infections in lymphoma and leukaemia patients receiving remission induction chemotherapy, in a double blind placebo controlled trial.

### Patients and methods

Forty-one patients with high grade non-Hodgkin lymphoma or acute lymphoblastic leukaemia who were to receive chemotherapy using the VAP regimen (Blackledge *et al.*, 1980) (Vincristine 2 mg intravenously weekly for six weeks, Adriamycin 60 mg m$^{-2}$ intravenously fortnightly for three doses, and oral prednisolone at 50 mg day$^{-1}$ for six weeks) were eligible for the trial. After obtaining informed consent patients were randomised to receive acyclovir 200 mg four times daily or placebo tablets of identical appearance. The tablets commenced on the first day of chemotherapy and were prescribed for six weeks.

Patients with non-Hodgkin lymphoma attended the outpatient clinic for examination and treatment each week, and those with acute leukaemia were inpatients for the duration of therapy. Throat swabs and blood samples for haematology, biochemistry and acyclovir levels were taken weekly. Blood for viral serology was taken every three weeks. At each outpatient visit patients were asked about oral symptoms and coldsores. Lesions present at the time of consultation were swabbed for viral culture and if applicable samples were taken from electron microscopy. Patients were seen three–four weeks after cessation of chemotherapy for repeat blood tests, viral throat swabs and clinical examination.

### *Virus isolation*

Swabs were taken into virus transport medium and subsequently inoculated into MRC5 human fibroblast and BK tissue cultures. Cultures were examined daily for three weeks for cytopathic effects.

### *Electron microscopy*

Material from lesions was placed upon microscope

Correspondence: H. Anderson.
Received 21 February 1984; accepted 26 March 1984.

© The Macmillan Press Ltd., 1984

slides and air dried. This was later resuspended in distilled water and a drop placed on a formvar-carbon support film on an EM grid. The film was then stained with 3% PTA and examined under the electron microscope. Photographs were taken of all positive samples.

*Serology*

Sera were tested by standard complement fixation techniques for antibodies to herpes viruses.

*Statistical design and analysis*

The design was double blind to prevent patient or observer bias. The number of patients entered was chosen to give a high power (90%) of detecting a real difference in the frequency of herpes simplex infection of 30–40%, in one-sided binomial tests at the 5% level of significance. The time to clinical infection and viral isolation in each trial arm was compared using a logrank test (Pete *et al.*, 1977), and results confirmed by Fisher's exact test (Siegel, 1956). Biochemical and haematological values were compared using analysis of variance.

### Results

Forty-one patients were entered into the trial. One patient, who died on the first day of the study, has been excluded from the analysis. Two patients were withdrawn – one refused to take further tablets on the eighth day of the trial because her mouth was sore due to candida (acyclovir), the other patient was withdrawn from treatment because he developed haemorrhagic cystitis (placebo). These two patients were included in the analysis until the acyclovir was discontinued. The other 38 patients had all received their full quota of VAP chemotherapy and acyclovir.

**Table I** Patient characteristics

| | *Acyclovir* | *Placebo* | *Total* |
|---|---|---|---|
| Number | 20 | 20 | 40 |
| Age median | 55 | 51 | 55 |
| range | 17–67 | 19–75 | 17–75 |
| Sex male | 14 | 11 | 25 |
| female | 6 | 9 | 15 |
| Lymphoma | 20 | 15 | 35 |
| Leukaemia | 0 | 5 | 5 |
| HSV titre < 1/10 at onset | 6 | 4 | 10 |

Twenty patients were randomised to acyclovir and 20 to placebo. Patient characteristics were similar for each treatment group, except that all five patients with acute lymphoblastic leukaemia were randomised to placebo (Table I). Twenty patients recalled previous HSV infection (seven received acyclovir).

Only one of the 20 patients in the acyclovir arm developed clinical infection – the patient described a coldsore that developed between hospital visits and lasted three days, whereas 12 of the 20 patients on placebo developed clinical infection ($P<0.001$). (Table II). The time to clinical infection for all patients was analysed by the logrank method and the results are shown in Figure 1. Five patients developed coldsores, three stomatitis, one patient developed oral ulceration, and another had oral ulceration, coldsores and pyrexia. Another patient

**Table II** Number of clinical infections and viral isolates during trial

| | *Acyclovir* | *Placebo* | *P value* |
|---|---|---|---|
| Clinical HSV during trial | [a]1/20 | 12/20 | <0.001 |
| Viral isolates during trial | [b]1/20 | 14/20 | <0.001 |
| Clinical HSV during trial in patients with HSV titre >1/10 | [a]1/14 | 10/16 | =0.004 |
| Viral isolates during trial in patients with HSV titre >1/10 | [b]1/14 | 12/16 | <0.001 |
| Clinical HSV during trial leukaemics excluded | [a]1/20 | 9/15 | <0.001 |
| Viral isolates during trial leukaemics excluded | [b]1/20 | 10/15 | <0.001 |

[a]Patient 35 clinical infection only, cultures not taken as patient was in out-patient phase.

[b]Patient 1 asymptomatic, viral culture positive on day one of trial and negative day 8.

**Figure 1** Kaplan–Meier curve of the time taken to develop clinical HSV infection during the trial of oral acyclovir prophylaxis. All 40 patients analysed.

had prolongued pharyngitis, laryngitis and a tongue ulcer. The final patient had coldsores and pyrexia.

Only one of the 20 patients in the acyclovir arm had a viral isolate. The patient was asymptomatic when HSV was isolated from the throat swab on the first trial day. The virus was not recovered from the day eight swab. However 14 patients on placebo had viral isolates during the trial ($P<0.001$). (Table II). Four isolates were from coldsores, seven from throat swabs; one from coldsores and ulcers, one was from a tongue swab, and one from oral ulcers.

Ten patients presented with HSV titres $<1/10$ (Table I). Patients with low titres have been excluded from other trials because the patients experience less HSV reactivation (4). If these patients are excluded there is still a statistically significant difference between the two groups in the number of clinical infections and viral isolates (Table II).

All five patients with acute leukaemia were randomised to placebo. Three developed clinical HSV, one of these three had a presenting HSV titre of $<1/10$. Four patients had HSV isolates, one with a presenting HSV titre of $<1/10$. Patients with leukaemia may be more immunosuppressed than lymphoma patients. If patients with leukaemia are excluded from analysis acyclovir still significantly reduces the incidence of clinical HSV infection and the number of viral isolates (Table II).

### *Deaths during the trial*

Four patients died during the trial, three were on acyclovir and one on placebo. Two men died unexpectedly and coroners post mortems showed no evidence of lymphoma death being due to myocardial infarction in one (died day 53) and small bowel performation in the other (died day ten). One man died of septicaemia and renal failure due to lymphoma obstructing the ureters (day ten). None of these deaths are thought to be acyclovir-related. The fourth patient was on placebo. She had leukaemia and died of fungal septicaemia despite treatment with Amphotericin B (day 31).

### *Infection after cessation of acyclovir*

After the trial period of six weeks there were 16 patients evaluable in the acyclovir group, and 18 in the placebo group (owing to the four deaths during the trial and the two withdrawls). Three patients on acyclovir and four on placebo developed clinical HSV (three with coldsores, three with oral ulceration and stomatitis, and one with atypical pneumonia who was excreting HSV in throat swabs and whose HSV titres rose from 1/20 to 1/360 during the infection). All these patients had presenting HSV titres of $>1/10$ and had lymphoma. There were seven patients with viral isolates after the trial (three in acyclovir arm and four on placebo). Six of them had clinical infection, and one had HSV isolated from throat swabs when asymptomatic. One patient with a healing coldsore had no viral isolates on EM or culture.

### *Treatment with intravenous acyclovir*

Two leukaemic patients received intravenous acyclovir during the trial (they continued the randomised tablets). Both were found to be receiving placebo when the trial was analysed. One patient had coldsores, oral ulceration and pyrexia. He had an initial HSV titre of $<1/10$ and this rose to 40 on day 38. He had five days acyclovir at $5\,mg\,kg^{-1}$ tds. The temperature settled at 48 h, the coldsore scab came off at three days, and the oral ulcers had healed at 5 days. The other was a lady with a pyrexia, confusion and eventually coma. She was on broad spectrum antibiotics and had platelet support. HSV was isolated in throat swabs just before the confusion developed. In case this was due to HSV encephalitis, IV acyclovir was commenced. There was no response. The patient died of fungal septicaemia.

### *HSV serology during the trial*

Only six patients had a fourfold rise in titres to HSV during the trial. Two had clinical infection and viral isolates, two had viral isolates only (one with atypical pneumonia may have had a viral pneumonia), one patient had a coldsore without viral isolation, and one patient had neither clinical infection nor viral isolate.

### *Acyclovir levels*

The ED50 for acyclovir is around 0.1 $\mu$M for HSV type 1 (Schaeffer *et al.*, 1978). During the trial therapeutic acyclovir levels for HSV were obtained. The peak acyclovir levels (at 1–2 h after the last dose) were between 1.59–8.63 $\mu$M, showing wide subject variation. There was no evidence of acyclovir accumulation. The patient who died of renal failure and septicaemia had acyclovir levels of 8.63 $\mu$M (2 h post dose) on the day prior to death. The female patient who developed a coldsore at home whilst on acyclovir tablets had levels of 0.47 $\mu$M six hours post dose the visit prior to the development of the coldsore and 0.71 $\mu$M five hours post dose the week later.

### In vitro *testing for acyclovir resistance*

All virus isolated during and after the trial has been stored frozen. After the trial was analysed isolates from the four patients in the acyclovir arm have been tested for acyclovir resistance using a plaque reduction assay, and none found. (One patient had isolates during the trial, and three after cessation of acyclovir). The ED50 values were all less than 0.1 $\mu$M, whereas the ED50 for a laboratory selected thymidine kinase negative mutant resistant to acyclovir was 7.19 $\mu$M.

### *Analysis of haematology and biochemistry results*

When the acute leukaemia patients are excluded (all randomised to placebo), the statistical analysis of haematology and biochemistry results showed that the only difference between the two groups was that the mean value of the blood urea was higher in the acyclovir group on day seven (8.14 mmol $l^{-1}$) compared to the placebo group (6.22 mmol $l^{-1}$) $P = 0.047$. However, there was no significant difference for serum creatinine between the two groups.

### Discussion

In this study acyclovir has significantly reduced the number of clinical infections and viral isolates due to HSV in patients with high grade non-Hodgkin lymphoma receiving VAP chemotherapy. There was no acyclovir associated toxicity during the trial. One patient, who has withdrawn from the trial because of haematuria, had previous cyclophosphamide-induced haematuria. As haematuria has been described in a patient treated with acyclovir (A. Clarke, Wellcome Foundation, personal communication) the patient was withdrawn from the trial. When the code was broken, the patient was found to be in the placebo arm.

*In vitro* acyclovir resistance was not observed in the viral isolate from the patient on day one of the trial, or from the three viral isolates from patients after the acyclovir was discontinued. This shows that aquired acyclovir resistance did not occur in these few patients. The patient who developed a coldsore between hospital visits had acyclovir levels above the ED50 for HSV before and after the episode of infection. As she denied non-compliance at the time of infection, and the coldsore was not witnessed one cannot comment on the reason why infection occurred.

This trial does not answer the question of whether acyclovir prophylaxis is better than prompt therapy of HSV infection with oral acyclovir. Although the latter would cost less, the patient would still experience the morbidity associated with infection.

In conclusion, this trial has shown that 200 mg oral acyclovir given six hourly is absorbed from the gastrointestinal tract, and serum levels adequate to prevent HSV in most patients are achieved. We have also confirmed the lack of toxicity shown by Gluckman *et al.* (1983). Acyclovir significantly reduced the incidence of clinical infection and viral isolates in non-Hodgkin lymphoma patients undergoing intensive chemotherapy with a VAP regimen.

Our grateful thanks go to all our collegues in the Manchester Lymphoma Group for allowing us access to their patients, and to the nursing staff of the medical oncology department. We thank the department of virology especially Mrs J. Lomax and Miss W. Bagguley for isolation and serology, Miss J. Roberts and Dr A. Curry for electron microscopy, and Dr J. Christophers for results on acyclovir resistance. Thanks go to the Wellcome Foundation for supply of tablets and Mrs A. Clark for initial help in setting up the trial.

### References

ASTON, D.L., COHEN, A. & SPINDLER, M.A. (1972). Herpesvirus hominis infection in patients with myeloproliferative and lymphoproliferative disorders. *Br. Med. J.*, **4**, 462.

BLACKLEDGE, G., BUSH, H., CHANG, J. & 9 others. (1980). Intensive combination chemotherapy with vincristine adriamycin and prednisolone (VAP) in the treatment of diffuse histology non-Hodgkin's lymphoma. *Eur. J. Cancer*, **16**, 1459.

CASAZZA, A.R., DUVALL, C.P. & CARBONE, P.P. (1966). Infection in lymphoma. *JAMA*, **197**, 118.

CHOU, S., GALLAGHER, J.C. & MERIGAN, T.C. (1981). Controlled clinical trial of intravenous acyclovir in heart-transplant patients with mucocutaneous herpes simplex infections. *Lancet*, **i**, 1392.

GLUCKMAN, E., LOTSBERG, J., DEVERGIE, A. & 6 others. (1983). Prophylaxis of herpes infections after bone-marrow transplantation by oral acyclovir. *Lancet*, **ii**, 706.

HANN, I.M., PRENTICE, H.G., BLACKLOCK, H.A. & 7 others. (1983). Acyclovir prophylaxis against herpes virus infections in severely immunocompromised patients: randomised double blind trial. *Br. Med. J.*, **287**, 384.

LAM, M.T., PAZIN, G.J., ARMSTRONG, J.A. & HO, M. (1981). Herpes simplex infection in acute myelogenous leukaemia and other haematologic malignancies: A prospective study. *Cancer*, **48**, 2168.

MEYERS, J.D., FLOURNOY, N. & THOMAS, E.D. (1980). Infection with herpes simplex virus and cell-mediated immunity after bone marrow transplant. *J. Infect. Dis.*, **142**, 338.

MITCHELL, C.D., BEAN, B., GENTRY, S.R., GROTH, K.E., BOEN, B.R. & BALFOUR, H.H. (1981). Acyclovir therapy for mucocutaneous herpes simplex in immunocompromised patients. *Lancet*, **i**, 1389.

MULLER, S.A., HERRMANN, E.C. & WINKELMANN, R.K. (1972). Herpes simplex infections in haematologic malignancies. *Am. J. Med.*, **52**, 102.

PETO, P., PIKE, M.C., ARMITAGE, P. & 7 others. (1977). Design and analysis of randomised clinical trials requiring prolonged observation of each patient. *Br. J. Cancer*, **35**, 1.

SARAL, R., BURNS, W.H., LASKIN, O.L., SANTOS, G.W. & LEITMAN, P.S. (1981). Acyclovir prophylaxis of herpes-simplex-virus infections. A randomised, double-blind, controlled trial in bone-marrow transplant recipients. *New Eng. J. Med.*, **305**, 63.

SCHAEFFER, H.J., BEAUCHAMP, L., COLLINS, P. & BAUER, D.J. (1978). 9-(2-hydroxyethoxymethyl) guanine activity against viruses of the herpes group. *Nature*, **272**, 583.

SIEGEL, S. (1956). The case of two independant samples. In *"Non-parametric statistics for the behavioural sciences"*. pp. 95–158. McGraw-Hill Book Co., NY.

WADE, J.C., NEWTON, B., McLAREN, C., FLOURNOY, N., KEENEY, R.E. & MEYERS, J.D. (1982). Intravenous acyclovir to treat mucocutaneous herpes simplex virus infection after bone marrow transplantation. A double blind trial. *Ann. Int. Med.*, **96**, 265.


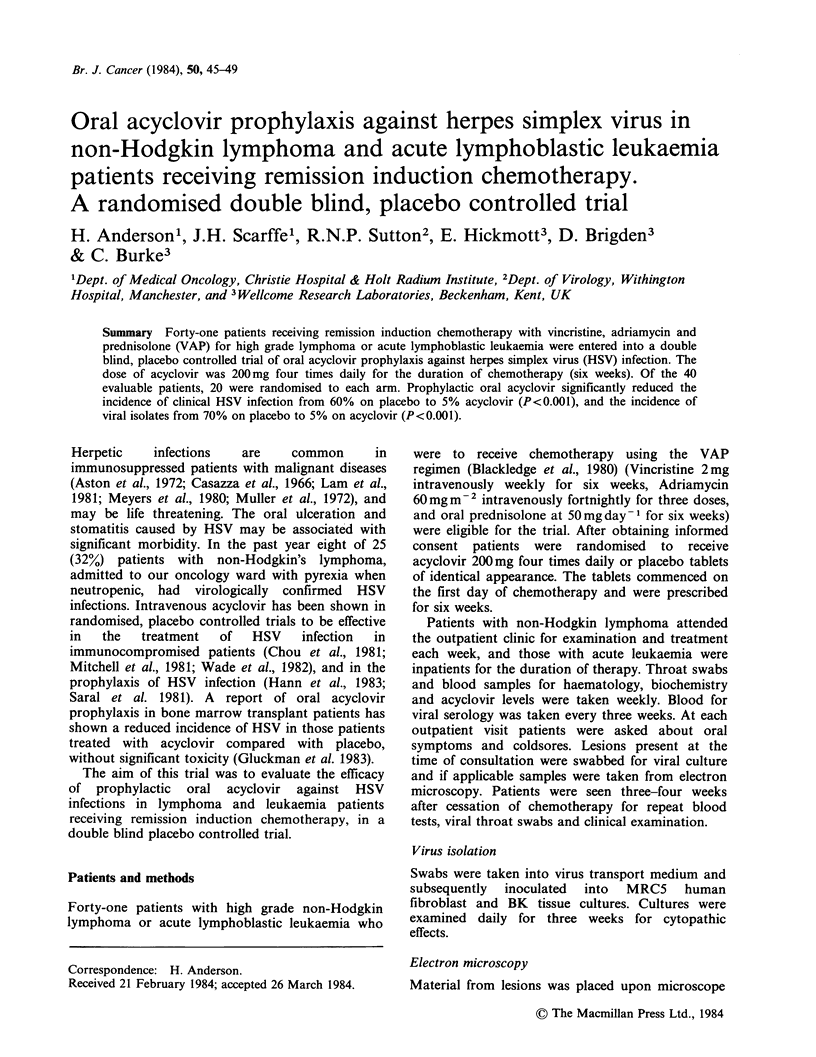

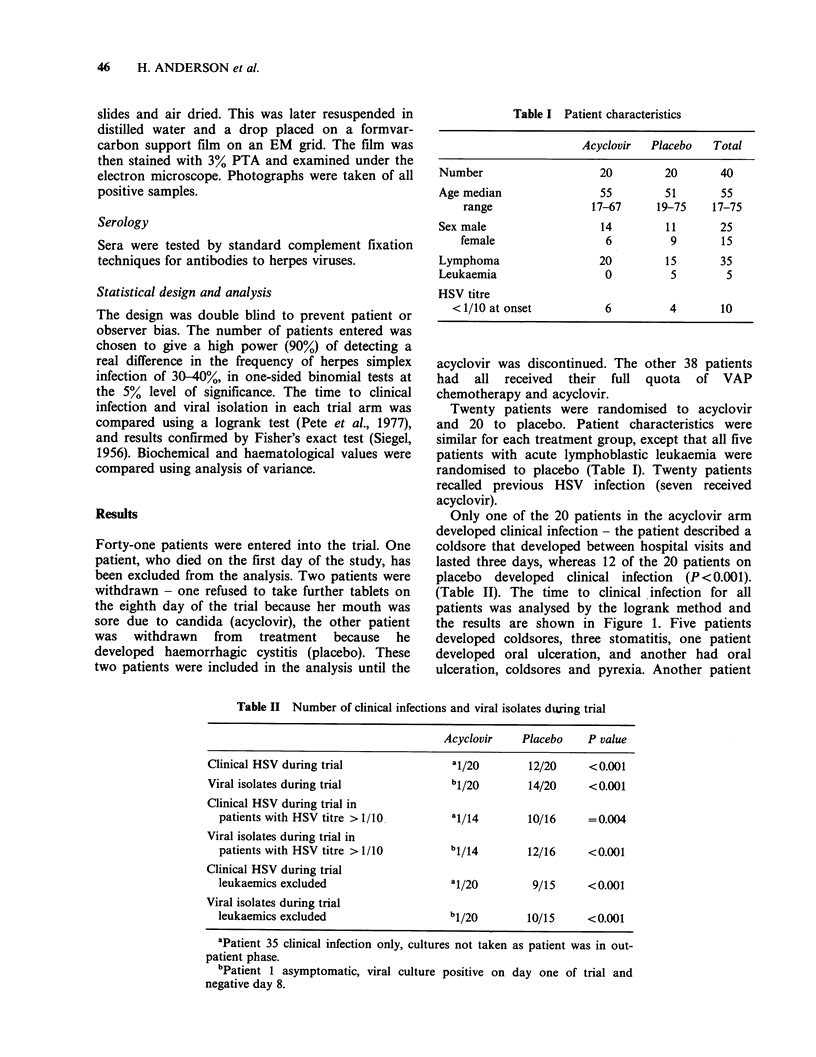

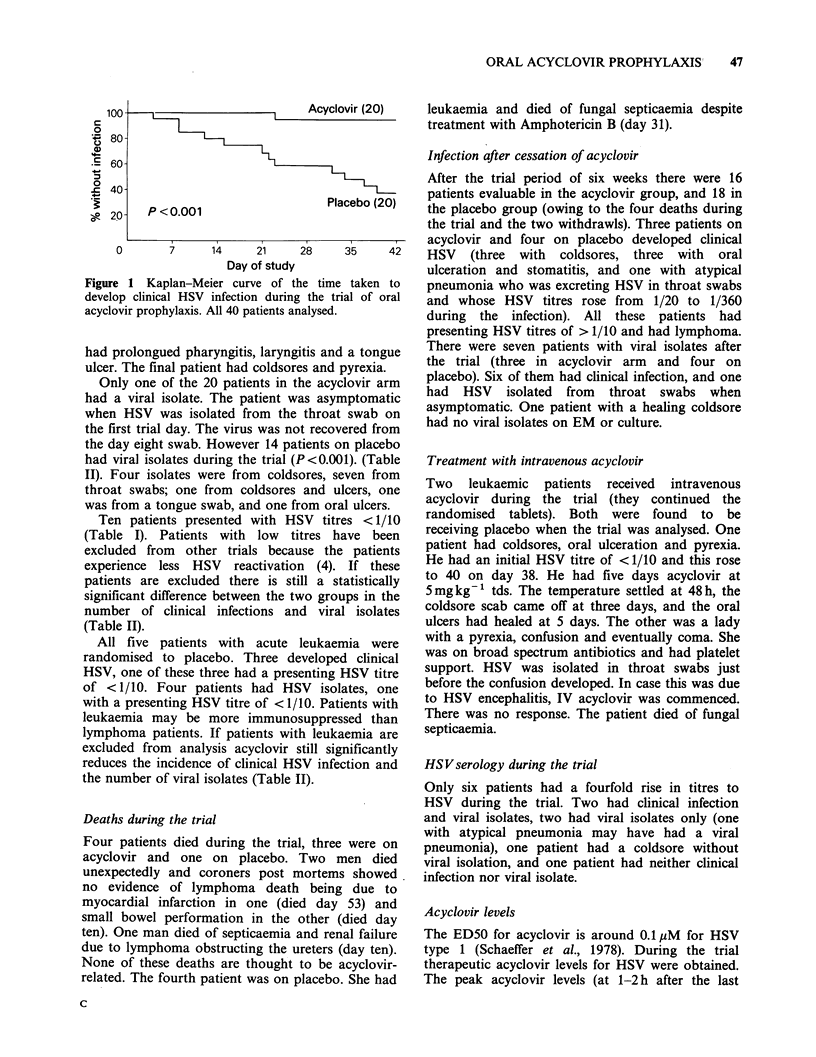

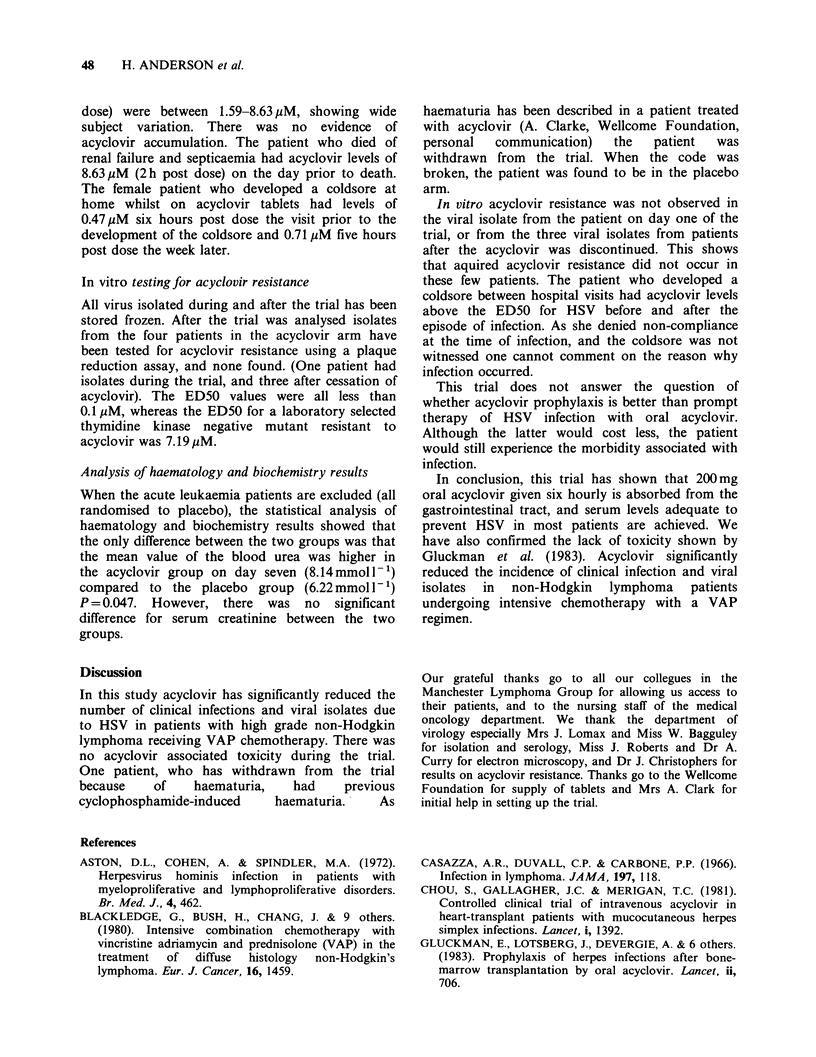

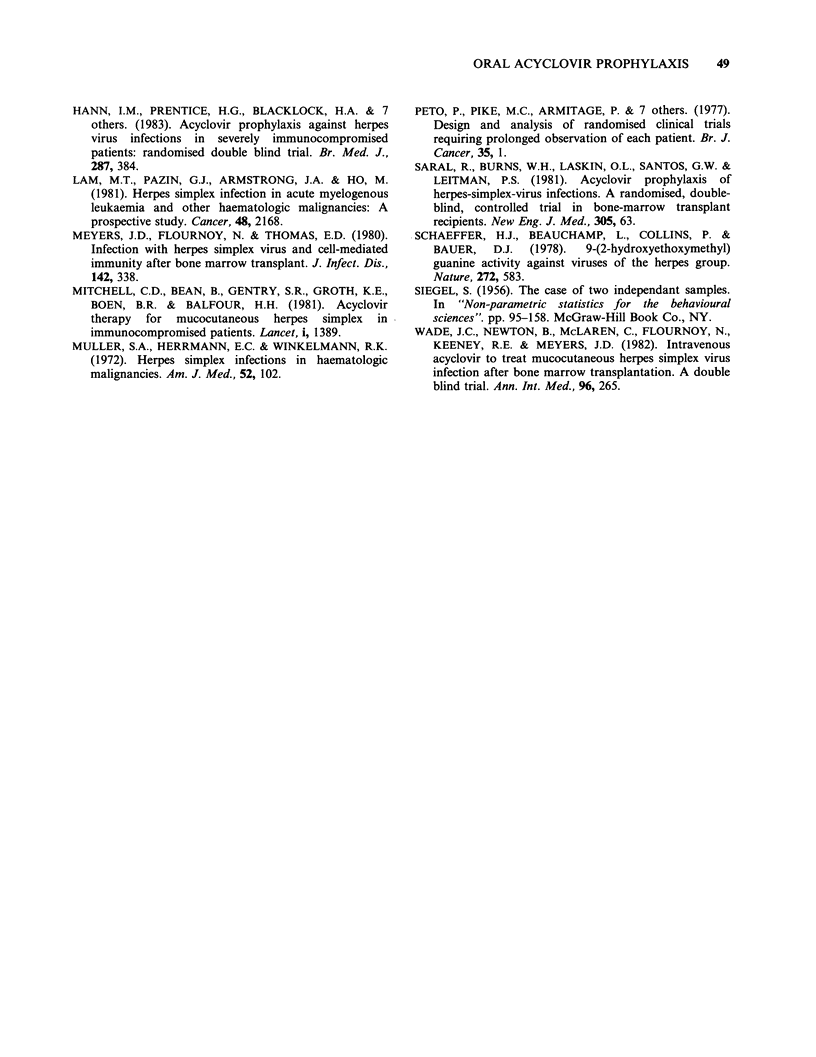


## References

[OCR_00448] Aston D. L., Cohen A., Spindler M. A. (1972). Herpesvirus hominis infection in patients with myeloproliferative and lymphoproliferative disorders.. Br Med J.

[OCR_00454] Blackledge G., Bush H., Chang J., Crowther D., Deakin D. P., Dodge O. G., Garrett J. V., Palmer M., Pearson D., Scarffe J. H. (1980). Intensive combination chemotherapy with vincristine, adriamycin and prednisolone (VAP) in the treatment of diffuse histology non-Hodgkin's lymphoma. (A report of 89 cases with extensive disease from the Manchester Lymphoma Group).. Eur J Cancer.

[OCR_00465] Chou S., Gallagher J. G., Merigan T. C. (1981). Controlled clinical trial of intravenous acyclovir in heart-transplant patients with mucocutaneous herpes simplex infections.. Lancet.

[OCR_00471] Gluckman E., Lotsberg J., Devergie A., Zhao X. M., Melo R., Gomez-Morales M., Nebout T., Mazeron M. C., Perol Y. (1983). Prophylaxis of herpes infections after bone-marrow transplantation by oral acyclovir.. Lancet.

[OCR_00479] Hann I. M., Prentice H. G., Blacklock H. A., Ross M. G., Brigden D., Rosling A. E., Burke C., Crawford D. H., Brumfitt W., Hoffbrand A. V. (1983). Acyclovir prophylaxis against herpes virus infections in severely immunocompromised patients: randomised double blind trial.. Br Med J (Clin Res Ed).

[OCR_00486] Lam M. T., Pazin G. J., Armstrong J. A., Ho M. (1981). Herpes simplex infection in acute myelogenous leukemia and other hematologic malignancies: a prospective study.. Cancer.

[OCR_00492] Meyers J. D., Flournoy N., Thomas E. D. (1980). Infection with herpes simplex virus and cell-mediated immunity after marrow transplant.. J Infect Dis.

[OCR_00498] Mitchell C. D., Bean B., Gentry S. R., Groth K. E., Boen J. R., Balfour H. H. (1981). Acyclovir therapy for mucocutaneous herpes simplex infections in immunocompromised patients.. Lancet.

[OCR_00504] Muller S. A., Herrmann E. C., Winkelmann R. K. (1972). Herpes simplex infections in hematologic malignancies.. Am J Med.

[OCR_00461] Nealon T. F., Sugerman H., Shea W., Fleegler E. (1966). An extracorporeal device to treat barbiturate poisoning. Use of anion-exchange resins in dogs.. JAMA.

[OCR_00515] Saral R., Burns W. H., Laskin O. L., Santos G. W., Lietman P. S. (1981). Acyclovir prophylaxis of herpes-simplex-virus infections.. N Engl J Med.

[OCR_00522] Schaeffer H. J., Beauchamp L., de Miranda P., Elion G. B., Bauer D. J., Collins P. (1978). 9-(2-hydroxyethoxymethyl) guanine activity against viruses of the herpes group.. Nature.

[OCR_00533] Wade J. C., Newton B., McLaren C., Flournoy N., Keeney R. E., Meyers J. D. (1982). Intravenous acyclovir to treat mucocutaneous herpes simplex virus infection after marrow transplantation: a double-blind trial.. Ann Intern Med.

